# Long-Term Survival after Extended Sleeve Lobectomy (ESL) for Central Non-Small Cell Lung Cancer (NSCLC): A Meta-Analysis with Reconstructed Time-to-Event Data

**DOI:** 10.3390/jcm12010204

**Published:** 2022-12-27

**Authors:** Dimitrios E. Magouliotis, Prokopis-Andreas Zotos, Anna P. Karamolegkou, Evangelos Tatsios, Kyriakos Spiliopoulos, Thanos Athanasiou

**Affiliations:** 1 Department of Cardiothoracic Surgery, University of Thessaly, Biopolis, 41110 Larissa, Greece; 2 Department of Anesthesiology, Hippocration General Hospital of Athens, 41500 Athens, Greece; 3 Department of Surgery and Cancer, Imperial College London, St Mary’s Hospital, London W2 1NY, UK

**Keywords:** lung cancer, NSCLC, extended sleeve lobectomy, ESL, pneumonectomy

## Abstract

Objective: We conducted a thorough literature search on patients with central non-small cell lung cancer (NSCLC) undergoing either extended sleeve lobectomy (ESL) or pneumonectomy (PN). Methods: We identified all original research studies that compared the long-term survival of ESL versus PN from 1990 to 2022. The primary endpoints were the median overall survival (OS) and disease-free survival (DFS). Complications, operative mortality, and the reoperation rate were the secondary endpoints. Regarding the primary endpoints, independent patient data were extracted from the included studies, and pooled Kaplan–Meier curves were constructed. A sensitivity analysis was performed using the leave-one-out method. Results: Nine studies were included in the qualitative and seven in the quantitative synthesis, including 431 patients. Patients in the ESL group demonstrated a significantly higher OS compared with the PN group (HR, 0.63; 95% CI, 0.46–0.87; *p* = 0.005). In addition, patients undergoing ESL presented a significantly higher DFS compared to the PN group (HR, 0.57; 95% CI, 0.40–0.80; *p* = 0.004). These findings were further validated with a sensitivity analysis. The most common complications in the ESL group were bronchopleural fistula (4.6%), stricture (3.1%), prolonged air leakage (7.3%), sputum retention (4.6%), pneumonia (7.7%), and pulmonary vein thrombosis (1.5%). ESL was associated with a low reoperation rate (1.5%) and operative mortality (1.2%). Conclusions: The present meta-analysis indicates that ESL is associated with enhanced survival outcomes compared to PN for patients with central NSCLC. Further randomized controlled trials are necessary to validate our findings.

## 1. Introduction

Surgical resections for centrally located non-small cell lung cancer (NSCLC) are associated with massive parenchymal extirpation and poor survival outcomes due to the highly aggressive nature of the disease, along with interlobar and mediastinal lymph nodal diaspora [1]. Pneumonectomy (PN) represents the traditional surgical approach for patients with centrally located tumors, leading to a substantial decline in lung function and quality of life, thus precluding adjuvant treatment or the further resection recurrence of the disease [2]. Consequently, PN is associated with certain restrictions in the treatment pathway, along with significant postoperative morbidity. To face this challenge, several approaches have been described by departments that have implemented the bronchovascular sleeve resection as an aggressive lung-preserving alternative to PN [1,2,3,4]. The classic bronchoplastic procedure for NSCLC includes the reconstruction of the pulmonary artery (PA), which has superior short- and long-term outcomes compared to those of PN [1,2]. In this context, the implementation of bronchoplastic procedures has exceeded that of PNs in patients with centrally located NSCLC, thus leading to an increasing ratio of sleeve lobectomies compared with PNs [2,3]. 

The classic sleeve lobectomy involves the resection of one lobe with an end-to-end bronchial anastomosis. Nonetheless, the management of centrally located NSCLC tumors may require the resection of more than one lobe, along with airway anastomoses in segmental bronchi and pulmonary vascular reconstructions [4,5]. The extended sleeve lobectomy (ESL) represents an atypical bronchoplasty with resections of more than one lobe and, consequently, is a more technically demanding procedure. Nonetheless, ESL has certain theoretical advantages, leading to its proposal as an alternative approach to PNs. In fact, although there is a significant interest in ESL as a treatment for centrally located NSCLC, there is limited available evidence comparing its survival, perioperative, and oncologic outcomes with PN. Therefore, the purpose of the present study is to summarize the existing data in the literature by comparing the survival and perioperative outcomes of ESL and PN for centrally located tumors and to provide the best up-to-date and currently available level of evidence on the topic.

## 2. Materials and Methods

### 2.1. Search and Articles Selection Strategy

The current meta-analysis was designed in accordance with the protocol agreed upon by all authors and the Preferred Reporting Items for Systematic Reviews and Meta-Analyses [6]. A systematic literature search was performed in three databases: (1) Pubmed (Medline), (2) Scopus (ELSEVIER), and (3) Cochrane Central Register of Controlled Studies (CENTRAL) (last search: 30 August 2022). The following terms were used in all possible combinations: “extended sleeve lobectomy”, “esl”, “pneumonectomy”, “pn”, “non-small cell lung cancer”, “nsclc”, “lung cancer”, “central”, “centrally” and “centrally located”. Inclusion criteria were (1) original reports with >10 patients, (2) published from 1990 to 2022, (3) written in English, (4) conducted on human subjects, and (5) reporting outcomes of patients undergoing ESL or PN for centrally located NSCLC. We excluded all duplicate articles and hand-searched the reference lists of all articles that were included for additional studies. Two independent reviewers (DEM, PAZ) extracted data from the included studies. Any potential discrepancies between the two investigators regarding the inclusion/exclusion of the selected studies were discussed with a senior author (TA) to incorporate only the articles that best matched the criteria until a consensus was reached. 

**Figure 1:**
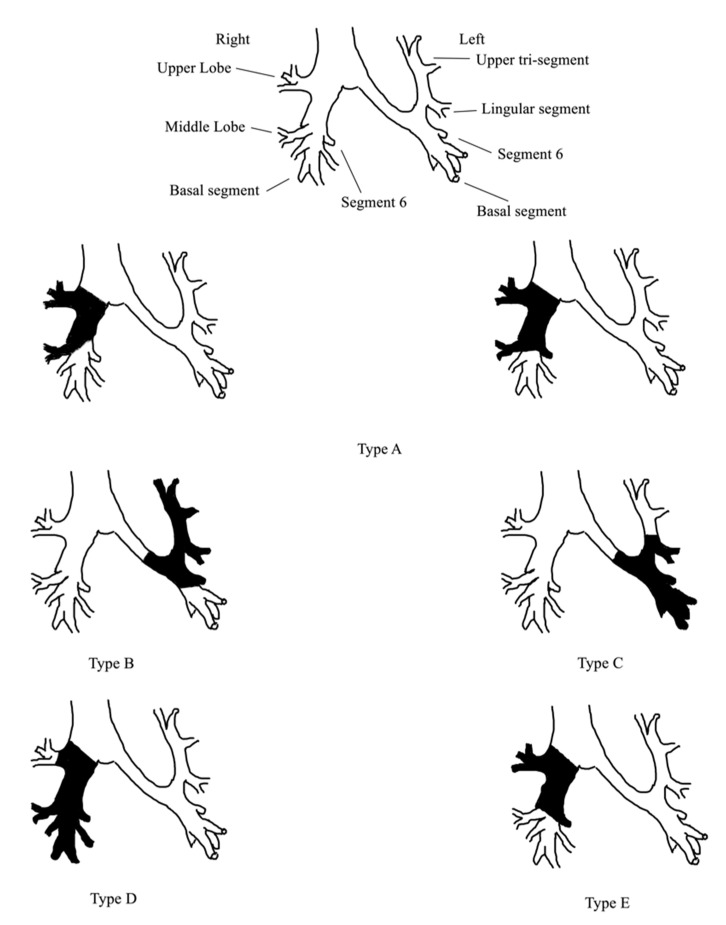
Different extended sleeve lobectomy (ESL) types according to the modified Okada classification.

Regarding the ESL procedure, we classified the surgical protocol that was employed in every study according to the Okada classification system [4] with only limited modifications. In their original paper [4], Okada et al. classified three types of ESL procedures: type A, including a resection of the right upper (RUL) and middle lobe (RML) with or without segment 6 (S6) resection combined with a reconstruction between the right main bronchus and the lower lobe (RLL) or basal segment bronchus; type B, including a resection of the left upper lobe and S6 with a reconstruction between the left main bronchus and the basal segment bronchus; type C, including a resection of the left lower lobe and lingular segment combined with a reconstruction between the segmental bronchus of the upper tri-segments and the left main bronchus. In the present study, we followed the modified Okada classification that also includes (i) the resection of the RML and the RLL with a reconstruction between the upper lobe bronchus and the right main bronchus and (ii) the resection of the RUL and S6 with a reconstruction between the right main bronchus and an orifice of the RML and basal segment. The latter are classified as types D and E of the modified classification we employed. We demonstrate each type of ESL procedure in Figure 1, according to the modified Okada classification. 

### 2.2. Data Extraction and Endpoints

For every included study, we extracted data relative to demographics (number of patients, gender, age, histology type, stage of disease, induction therapy) and the type of ESL procedure (A, B, C, D, or E), along with survival and perioperative endpoints (overall survival (OS), disease-free survival (DFS), complications, reoperation rate, and operative mortality). In cases where more than one study analyzed the same population, only the study with the largest sample or the longest follow-up was included in the meta-analysis. 

Median OS and DFS were the primary endpoints. Complications, reoperation rate, and operative mortality were the secondary endpoints. Prior to the survival analyses, we performed a chi-square analysis regarding the patients’ staging to ensure that both groups have similar oncologic characteristics and to limit potential bias. Pooled survival analysis of OS was conducted using the published Kaplan–Meier graphs from the included studies, employing the two-stage approach, as was previously described [7]. At first, raw data coordinates (time, survival probability) were extracted for each arm in every Kaplan–Meier curve. Secondly, the data coordinates extracted at the first stage were processed in conjunction with the patients at risk at certain predefined time points, and individual patient data (IPD) were reconstructed. The final step was to pool the reconstructed independent patient data from all treatment arms, along with visualizing them in a Kaplan–Meier graph. Moreover, we employed the Gehan–Breslow–Wilcoxon test to compare the OS and DFS between the ESL and PN groups. A *p*-value <0.05 was set as the threshold, indicating a statistically important result. Finally, we employed the Mantel–Haenszel statistical method to estimate the hazard ratio (HR) with its 95% confidence interval (95% CI). 

### 2.3. Sensitivity Analysis on Survival Endpoints

Aiming to further validate our findings, we conducted additional sensitivity analyses regarding OS and DFS using the leave-one-out method. The leave-one-out method involves performing a meta-analysis on each subset of the studies obtained by leaving out exactly one study. 

### 2.4. Quality and Publication Bias Assessment 

To evaluate the quality appropriateness of the included non-RCTs, we employed the Newcastle–Ottawa Scale (NOS) [8]. The scale uses a range varying from 0 to 9 stars, and studies with a score equal to or higher than five stars were considered to be of adequate methodological quality. The Risk of Bias in Non-Randomized Studies of Interventions tool (ROBINS-I) was employed to evaluate the included studies for risk of bias [9]. No RCTs were identified/included regarding this topic. Two reviewers (DEM, PAZ) rated the studies independently and discrepancies were discussed until a consensus was reached. 

## 3. Results

### 3.1. Search Strategy and Patient Demographics

**Figure 2:**
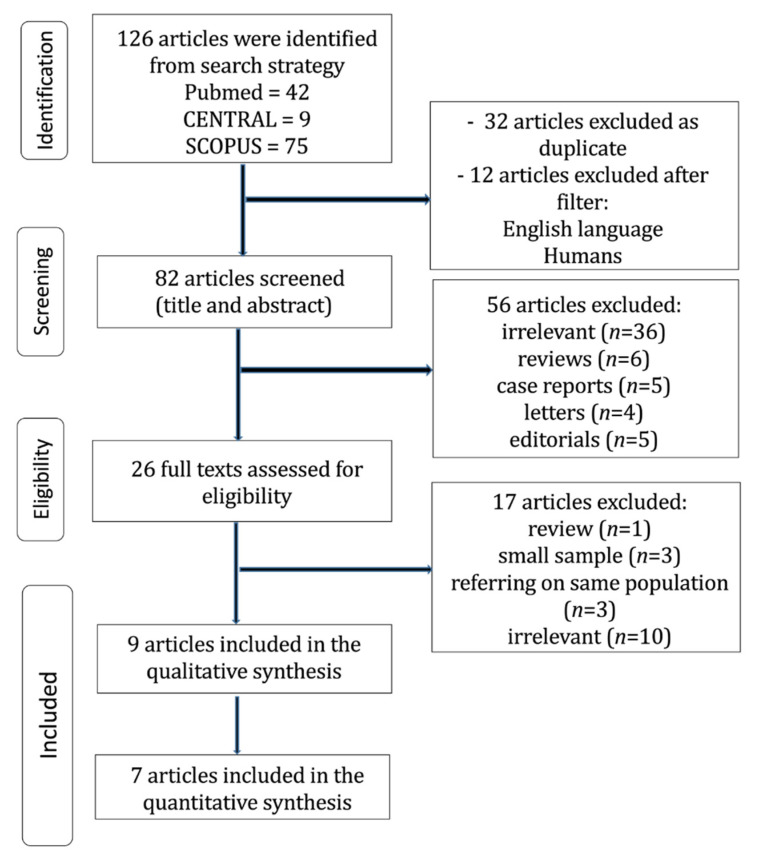
Trial flow of the current meta-analysis.

The flow diagram regarding the search strategy is provided in Figure 2, and the Prisma Checklist is demonstrated in Table S1. The characteristics of the incorporated studies are demonstrated in Table 1. From the 126 articles that were retrieved originally, nine studies [4,10,11,12,13,14,15,16,17] were included in the qualitative and seven in the quantitative analysis [10,11,12,13,14,15,16]. The level of agreement between the reviewers was “almost perfect” (kappa = 0.844; 95% CI: 0.672, 1.000). In addition, the study design was prospective in four studies [10,12,15,16] and retrospective in five studies [4,11,13,14,17]. Moreover, the incorporated studies were conducted in Japan [4,11,13,17], France [10], Italy [12,15], Korea [14], and China [16], and were published between 1999 and 2022. The ESL and PN patient populations ranged from 15 to 63 and from 15 to 76 patients, respectively. The total study population was 431 patients; 259 patients underwent ESL and 172 patients underwent PN. The Okada classification was implemented in two studies [4,15] and the modified Okada classification in six studies [10,11,13,14,16,17]. There was one study [12] that did not classify the procedures. 

**Figure 3:**
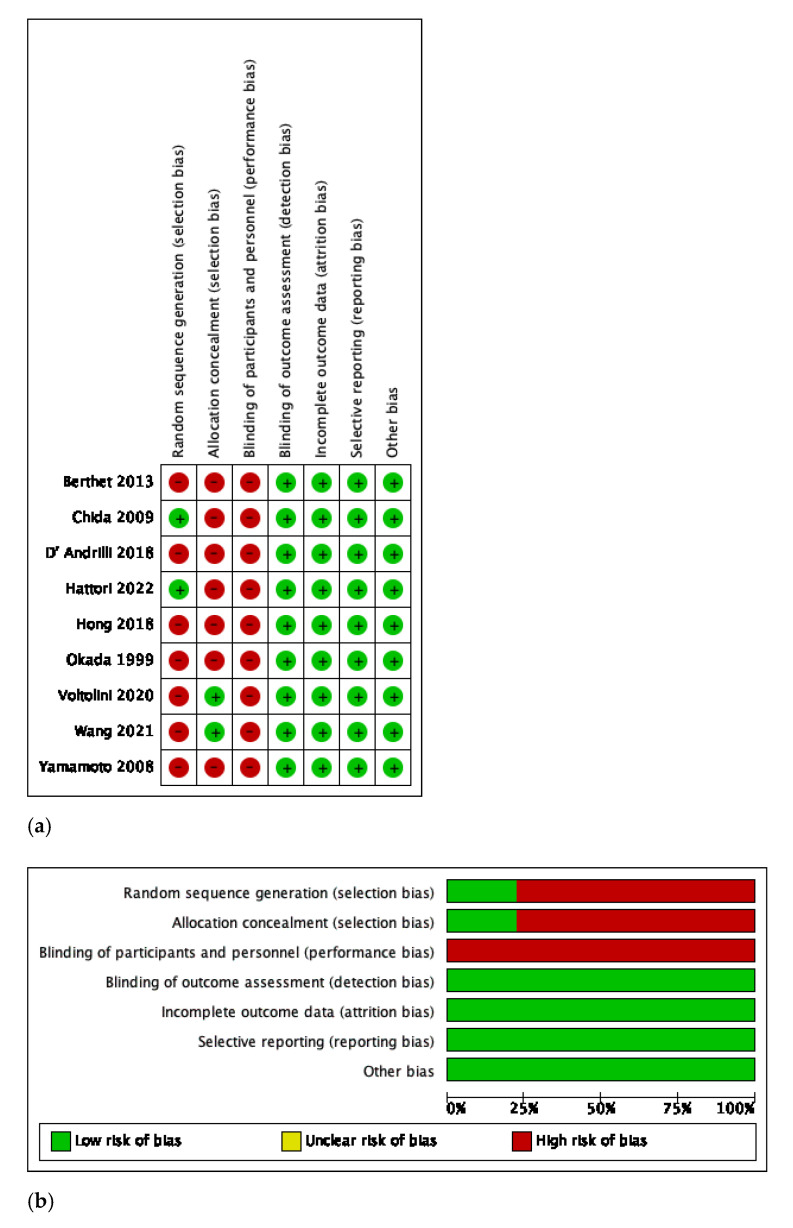
(**a**) Risk of Bias in Non-Randomized Studies of Interventions tool with traffic lights [4,10,11,12,13,14,15,16,17]. (**b**) Risk of Bias in Non-Randomized Studies of Interventions tool with summary plot.

The baseline characteristics of patients from each included study are demonstrated in Table 1 and complications in Table 2. The majority of the patients presented squamous cell carcinoma (SCC) and fewer presented adenocarcinoma (ADC). The R0 resection rate was 97.7% in the ESL group. The rate of induction treatment ranged from 5.7% to 41%. No cases using extracorporeal membrane oxygenation (ECMO) or any other type of complex support were described in the included studies. No difference was reported between the patients that underwent either ESL or PN regarding staging (*p* = 0.065). The NOS assessment of quality for all studies is shown in Table 1. Figure 3a,b demonstrates the qualitative assessment of the studies according to the ROBINS-I tool. The authors’ main concerns are mainly related to biases associated with the participants’ selection and performance.

### 3.2. Primary Endpoints: OS and DFS

**Figure 4:**
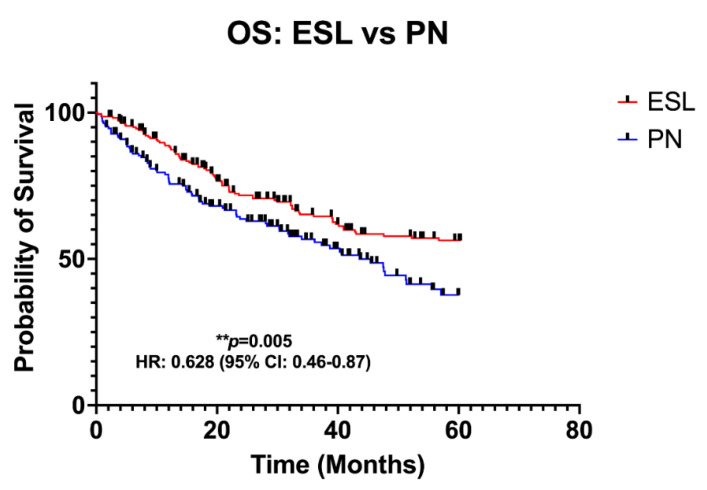
Pooled Kaplan–Meier curve for overall survival (OS) with extended sleeve lobectomy (ESL) compared to pneumonectomy (PN). CI, confidence interval; HR, hazard ratio. ** indicates significant difference.

**Figure 5:**
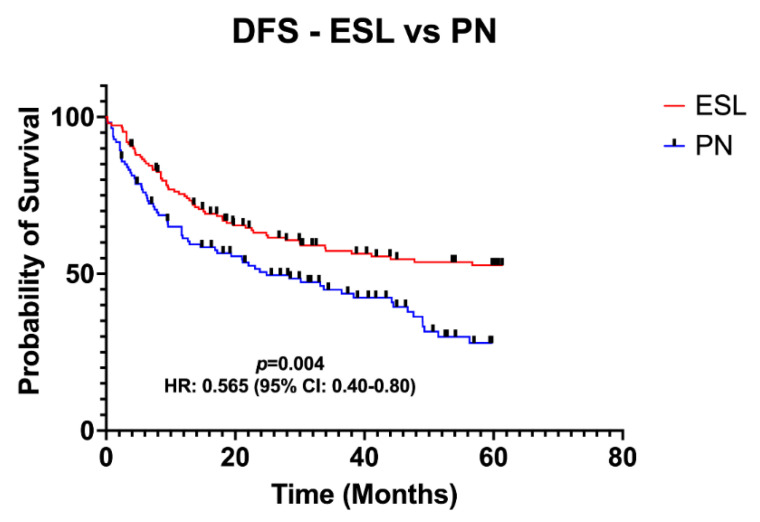
Pooled Kaplan-Meier curve for disease-free survival (DFS) regarding extended sleeve lobectomy (ESL) compared to pneumonectomy (PN). HR: hazard ratio; CI: confidence interval.

Figure 4 demonstrates the Kaplan–Meier survival curves regarding OS. The data of 388 patients (ESL: 222 patients; PN: 166 patients) from seven studies, with a median follow-up ranging from 3 to 222 months, were pooled. Patients in the ESL group presented a significantly higher OS (HR: 0.63; 95% CI: 0.46–0.87; *p* = 0.005). Figure 5 demonstrates the Kaplan–Meier survival curves regarding disease-free survival in the total population. The data of 263 patients (ESL: 150 patients; PN: 113 patients) were pooled. Patients in the ESL group were associated with a significantly higher DFS (HR: 0.57; 95% CI: 0.40–0.80; *p* = 0.004).

### 3.3. Secondary Endpoints: Complications, Operative Mortality, and Adjuvant Treatment

Despite our initial interest to further analyze and compare the complication rate between the two groups, this was not performed due to the limited available data. The most common complications of the ESL group are demonstrated in Table 2. These were prolonged air leakage (7.3%), postoperative pneumonia (7.7%), sputum retention (4.6%), atrial fibrillation (6.2%), and bronchopleural fistula (4.6%). The operative mortality was low (1.2%). All patients with pN2 staging were referred for adjuvant treatment; however, there was no available data to make any comparisons between the two groups regarding the number of patients that successfully completed the treatment.

### 3.4. Sensitivity Analysis 

No difference regarding the survival outcomes was found after performing the leave-one-out sensitivity analysis. All findings were in accordance with the total analysis of OS and DFS comparing ESL with PN.

## 4. Discussion

The current evidence provided by the literature on the benefits of ESL over PN for centrally located NSCLC remains limited, and there is no RCT available. In this context, the current meta-analysis represents the highest available level of evidence. In fact, there is no other meta-analysis available in the literature to the best of our knowledge. The present meta-analysis included nine articles comparing ESL and PN for central tumors using reconstructed time-to-event patient data. Given the great technical complexity of ESL compared with simple sleeve lobectomy and PN, adequate survival and oncologic outcomes should be demonstrated to counterbalance the perioperative risk. As a result, the survival outcomes were our primary endpoints. According to the outcomes of the present meta-analysis, ESL is associated with higher OS and DFS compared to PN. In the same context, ESL demonstrated a high rate of R0 resection, thus reaffirming the oncologic adequacy of the procedure. Moreover, the operative mortality was relatively low, with only three deaths (1.2%) reported in all the included articles. This outcome is similar to the mortality in patients undergoing sleeve lobectomy (SL) (1.3%) and lower than pneumonectomy (5.3%), as demonstrated in a large study including 1,230 patients [1]. Nonetheless, complications were not rare due to the high technical complexity of these procedures. Given the promising results of other treatment strategies, along with the induction treatment for advanced NSCLC, ESL has the potential to become a lung-sparing approach of choice for selected patients. Based on the promising outcomes of this strategy, more centers have tended to adopt it, a trend that explains the fact that most of the included studies were published during the last five years.

Different ESL procedures were employed in the included studies. As was previously commented, these are divided according to the modified Okada classification to types A–E, and each one has its own characteristics. Type A procedures require a long bronchial resection from the level of the right main to the basal segment bronchus. Consequently, the management to reduce the anastomosis-related tension is important to prevent anastomotic complications. In this context, type A ESL procedures frequently require a combined angioplasty of the pulmonary artery. The same principles regarding the extent of bronchial resection exist also in type B procedures. On the other hand, type C ESL has different technical characteristics, given that a size discrepancy might occur between the proximal and distal bronchial stump, thus highlighting the need for a careful caliber adjustment in anastomosis. These characteristics are also similar to type D ESL procedures. Due to these special traits, the extent of lung-sparing in both C and D procedures might be less compared with other types. Consequently, the meticulous management of the residual pleural space is crucial to preventing space-related postoperative morbidities, such as empyema and BPF. Perhaps the meticulous drainage of the chest cavity or the artificial phrenic nerve palsy might reduce the incidence of these complications [18]. Finally, the characteristics of the type E ESL procedure are similar to type A.

Due to their high technical complexity, ESL procedures are associated with a significant incidence of postoperative morbidity. However, the rate of reoperations was relatively low (1.5%) compared to SL (1.8%) and PN (23%), according to literature data [2]. Major complications are delayed air leakage, bronchial strictures, and BPFs. To reduce the tension of bronchial reconstruction in type A ESL procedures, a transposition of the inferior to the superior pulmonary vein or a PA reconstruction is often required. Owning to these operating maneuvers, vascular complications, such as PA thrombosis or the necrosis of the lung parenchymal remnant, are not rare [18]. According to our outcomes, the incidence of BPF and stricture was 4.6% and 3.1%, respectively. The incidence of BPF was higher compared with SL (1.8%) but significantly lower than pneumonectomy (14%), according to a previous study [2]. In addition, persistent air leakage was also relatively low (7.3%) compared to SL (12.7%) [2]. Furthermore, sputum retention was another relatively common complication according to our outcomes, which may require the endoscopic cleaning of airways to maintain good patency of the airways and lung expansion. An additional role of bronchoscopy is to evaluate the quality of the anastomosis prior to patient’s discharge [19]. Finally, PVT was another less frequent complication (1.5%) that can occur as a result of the overstretching of the pulmonary vein. The use of pericardial cutting has been proposed as a measure to prevent this complication in type A and B procedures [18]. 

Given the lack of randomized clinical trials comparing the feasibility of ESL over PN for centrally located NSCLC, the current work is the largest up-to-date comparative study, incorporating 431 patients. The present analysis supports the superiority of ESL in terms of long-term survival over PN for patients with central NSCLC. Nonetheless, due to the high complexity of these procedures, they should be performed by experienced thoracic surgeons in high-volume centers. Consequently, it is crucial to define the exact selection criteria for the best candidates to undergo ESL. In this context, the current meta-analysis provides the best currently available level of evidence that might help multidisciplinary decision-making on complex cases, given the lack of guidelines on the topic. The herein presented evidence should be taken into account during the composing of future guidelines on the management of central lung tumors. Nonetheless, our outcomes should be further validated by well-designed RCTs.

The limitations of the present study are mainly associated with the limitations of the included studies. Most of the studies were retrospective, and no RCT was identified through the literature search, thus posing a certain limitation in this study. Furthermore, the incorporated studies are related to biases related to participants’ selection and performance. In addition, the differences among institutions regarding the treatment protocols, selection criteria, and perioperative management pose several limitations. In the same context, the selection criteria were not homogenous and may have been based on the patients’ clinical attributes and status, thus posing a selection bias that could not be adjusted in the present study. Finally, patient data were gathered from Kaplan–Meier-derived data, and not from individual patient follow-ups, thus limiting the ability to perform further subgroup, multivariate, or propensity score matching analyses. 

On the other hand, the strengths of the present meta-analysis include (i) the clear literature search and data extraction protocol, (ii) the well-specified inclusion/exclusion criteria, (iii) the literature search in three databases, (iv) the quality assessment of the included studies, (v) the detailed presentation of the outcomes, (vi) the extraction of survival data at the level of the independent patient, and (vii) the performance of sensitivity analyses. 

## 5. Conclusions

In the context of patients with central NSCLC undergoing surgery, ESL seems to provide adequate outcomes in terms of median OS and DFS, an outcome that should be further validated by future well-designed studies with a greater population. This evidence should be used as an adjunct to the available guidelines during the multidisciplinary discussion of complex cases. Nonetheless, due to the high complexity of these procedures, they should be performed by experienced surgeons in high-volume centers, and clear selection criteria should be defined. Further randomized controlled trials are needed to confirm or refute the authors’ current findings.

## Figures and Tables

**Table 1:** Baseline characteristics of the studies and patients that were included in the meta-analysis.

Study ID, Year	Country	Study Design	Patients, n		Mean Age, y ± SD		Histology, n (%)		Pathologic Stage, n (%)		Neoadjuvant Therapy, n (%)		Type of Procedure, n (%)	Combined Angioplasty, n (%)	Follow-up, m	NOS
			ESL	PN	ESL	PN	ESL	PN	ESL	PN	ESL	PN				
Berthet 2013 [10]	France	P	27	-	62.7 ± 8.2	-	ADC: 12 (44) SCC: 15 (56)	-	I: 7 (26) II: 14 (52) III: 6 (22)	-	7 (25.9)	-	A:11 (41), B:7 (26), C:2 (7), D:2 (7), E:5 (19)	11 (40.7)	7–69	7
Chida 2009 [11]	Japan	R	23	15	65.36 ± 7.9	-	ADC: 4 (17) SCC: 18 (78) O: 1 (5)	-	I: 4 (17) II: 6 (26) III: 13 (57)	NR	7 (25.9)	-	-	11 (40.7)	3–36	6
D’ Andrilli 2018 [12]	Italy	P	24	-	60.4 ± 9.8	-	ADC: 12 (50) SCC: 8 (33) O: 4 (17)	-	I: 7 (29) II: 8 (33) III: 9 (38)	-	8 (33.3)	-	N/R	N/R	6–72	6
Hattori 2022 [13]	Japan	R	43	76	66.5 ± 10.1	N/R	ADC: 16 (37) SCC: 22 (51) O: 5 (12)	ADC: 21 (28) SCC: 45 (59) O: 10 (13)	I: 1 (2) II: 19 (44) III: 23 (54)	I: 1 (1) II: 25 (33) III: 50 (66)	5 (12)	13 (17)	A: 10 (23), B: n=8 (19), C: n=16 (37), D: n=9 (21)	20 (47)	6-222	6
Hong 2018 [14]	Korea	R	63	-	60 ± 10	-	ADC: 2 (3) SCC: 54 (86) O: 7 (11)	-	I: 19 (30) II: 30 (48) III: 14 (23)	-	4 (5.7)	-	A: 14 (22), B: 4 (6), C: 8 (13), D: 37 (59)	13 (20.6)	48	6
Okada 1999 [4]	Japan	R	15	-	64 ± 6	-	ADC: 2 (13) SCC: 13 (87)	-	I: 0 (0) II: 9 (60) III: 6 (40)	-	4 (27)	-	A: 6 (40), B: 4 (27), C: 5 (33)	8 (53.3)	9–106	6
Voltolini 2020 [15]	Italy	P	22	38	68 (51-79)	67 (51-83)	ADC: 8 (36.4) SCC: 14 (63.6)	N/R	I: 1 (4.5) II: 8 (36.3) III: 13 (59.1) IV: 0 (0)	I: 2 (5.2) II: 12 (31.6) III: 23 (60.5) IV: 1 (2.6)	7 (31.8)	12 (31.6)	A: 8 (36), B: 1 (5), C: 13 (59)	7 (31.8)	4-57	7
Wang 2021 [16]	China-Italy	P	22	43	54 ± 10	54 ± 9	SCC: 15 (68) O: 7 (32)	SCC: 31 (72) O: 12 (28)	*N0: 11 (50) N1: 6 (27) N2: 5 (23)	*N0: 22 (51) N1: 13 (30) N2: 8 (19)	9 (41)	18 (42)	A: 7 B: 10 C:2 D: 3	-	11-58	7
Yamamoto 2008 [17]	Japan	R	20	-	N/R	-	N/R	-	N/R	-	N/R	-	A: 2 (10), B: 8 (40), C: 7 (35), E: 3 (15)	9 (45)	13–113	5

Abbreviations: NOS = Newcastle–Ottawa Scale; R = Retrospective; P = Prospective; NR = Not Reported; ESL = Extended Sleeve Lobectomy; PN = Pneumonectomy; y = years; m = months; n = number; ADC = Adenocarcinoma; SCC = Squamous Cell Carcinoma; SD = Standard Deviation; NOS = Newcastle Ottawa Scale * Data relevant to pathologic N staging is provided.

**Table 2:** Summary of the main complications associated with extended sleeve lobectomy (ESL).

Complications	Number of Patients (259) *n*, (%)
Sputum retention	12 (4.6)
Pneumonia	20 (7.7)
Prolonged air leakage	19 (7.3)
Chylothorax	2 (0.8)
AF	16 (6.2)
BPF	12 (4.6)
Stricture	8 (3.1)
PVT	4 (1.5)
Reoperation	4 (1.5)
Operative mortality	3 (1.2)

Abbreviations: *n* = number; AF = Atrial Fibrillation; BPF = Bronchopleural Fistula; PVT = Pulmonary Vein Thrombosis.

## Data Availability

The data that support the findings of this study are available from the corresponding author, upon reasonable request.

## Supplementary Materials

The following supporting information can be downloaded at: https://www.mdpi.com/article/10.3390/jcm12010204/s1, Table S1: PRISMA 2020 Checklist.

## References

[1] Deslauriers J., Gregoire J., Jacques L.F., Piraux M., Guojin L., Lacasse Y. (2004). Sleeve lobectomy versus pneumonectomy for lung cancer: A comparative analysis of survival and sites or recurrences. Ann. Thorac. Surg..

[2] Gomez-Caro A., Garcia S., Reguart N., Cladellas E., Arguis P., Sanchez M., Gimferrer J.M. (2011). Determining the appropriate sleeve lobectomy versus pneumonectomy ratio in central non-small cell lung cancer patients: An audit of an aggressive policy of pneumonectomy avoidance. Eur. J. Cardiothorac. Surg..

[3] Shimizu H., Okada M., Toh Y., Doki Y., Endo S., Fukuda H., Hirata Y., Iwata H., Committee for Scientific Affairs, The Japanese Association for Thoracic Surgery (2021). Thoracic and cardiovascular surgeries in Japan during 2018: Annual report by the Japanese Association for Thoracic Surgery. Gen. Thorac. Cardiovasc. Surg..

[4] Okada M., Tsubota N., Yoshimura M., Miyamoto Y., Matsuoka H., Satake S., Yamagishi H. (1999). Extended sleeve lobectomy for lung cancer: The avoidance of pneumonectomy. J. Thorac. Cardiovasc. Surg..

[5] Rendina E.A., Venuta F., de Giacomo T., Rossi M., Coloni G.F. (2002). Parenchymal sparing operations for bronchogenic carcinoma. Surg. Clin. N. Am..

[6] Page M.J., McKenzie J.E., Bossuyt P.M., Boutron I., Hoffmann T.C., Mulrow C.D., Shamseer L., Tetzlaff J.M., Akl E.A., Brennan S.E. (2021). The PRISMA 2020 statement: An updated guideline for reporting systematic reviews. BMJ.

[7] Liu N., Zhou Y., Lee J.J. (2021). IPD from KM: Reconstruct individual patient data from published Kaplan-Meier survival curves. BMC Med. Res. Methodol..

[8] Stang A. (2010). Critical evaluation of the Newcastle-Ottawa scale for the assessment of the quality of nonrandomized studies in meta-analyses. Eur. J. Epidemiol..

[9] Sterne J.A., Hern an M.A., Reeves B.C., Savović J., Berkman N.D., Viswanathan M., Henry D., Altman D.G., Ansari M.T., Boutron I. (2016). ROBINS-I: A tool for assessing risk of bias in non-randomized studies of interventions. BMJ.

[10] Berthet J.P., Paradela M., Jimenez M.J., Molins L., Gómez-Caro A. (2013). Extended sleeve lobectomy: One more step toward avoiding pneumonectomy in centrally located lung cancer. Ann. Thorac. Surg..

[11] Chida M., Minowa M., Miyoshi S., Kondo T. (2009). Extended sleeve lobectomy for locally advanced lung cancer. Ann. Thorac. Surg..

[12] D’Andrilli A., Maurizi G., Ciccone A.M., Andreetti C., Ibrahim M., Menna C., Vanni C., Venuta F., Rendina E.A. (2018). Long-segment pulmonary artery resection to avoid pneumonectomy: Long-term results after prosthetic replacement. Eur. J. Cardiothorac. Surg..

[13] Hattori A., Matsunaga T., Fukui M., Takamochi K., Oh S., Suzuki K. (2022). Surgical Outcome After Extended Sleeve Lobectomy in Centrally Located Non-small Cell Lung Cancer. Ann. Thorac. Surg..

[14] Hong T.H., Cho J.H., Shin S., Kim H.K., Choi Y.S., Zo J.I., Shim Y.M., Kim J. (2018). Extended sleeve lobectomy for centrally located non-small-cell lung cancer: A 20-year single-centre experience. Eur. J. Cardiothorac. Surg..

[15] Voltolini L., Gonfiotti A., Viggiano D., Borgianni S., Farronato A., Bongiolatti S. (2020). Extended sleeve-lobectomy for centrally located locally advanced non-small cell lung cancer is a feasible approach to avoid pneumonectomy. J. Thorac Dis..

[16] Wang X., Jiang S., You X., Aramini B., Shabaturov L., Jiang G., Zhu Y., Fan J. (2021). Extended Sleeve Lobectomy is an Alternative for Centrally Located Lung Cancer with Superior Short- and Long-term Outcomes. Clin. Lung Cancer..

[17] Yamamoto K., Miyamoto Y., Ohsumi A., Kojima F., Imanishi N., Matsuoka K., Ueda M., Hamada C. (2008). Sleeve lung resection for lung cancer: Analysis according to the type of procedure. J. Thorac. Cardiovasc. Surg..

[18] Waseda R., Iwasaki A. (2018). Extended sleeve lobectomy: Its place in surgical therapy for centrally located non-small cell lung cancer and a review of technical aspects. J. Thorac. Dis..

[19] Ludwig C., Stoelben E. (2012). A new classification of bronchial anastomosis after sleeve lobectomy. J. Thorac. Cardiovasc. Surg..

